# Supplementary material for: HER2-Positive Gastroesophageal Cancers Are Associated with a Higher Risk of Brain Metastasis
Source: Cancers (Basel). 2022 Nov 23;14(23):5754. doi: 10.3390/cancers14235754 (PMC9735596; doi:10.3390/cancers14235754)
Supplement: Supplementary file 1 [file cancers-14-05754-s001.zip › cancers-1983452-supplementary.pdf]

Supplementary Figure S1

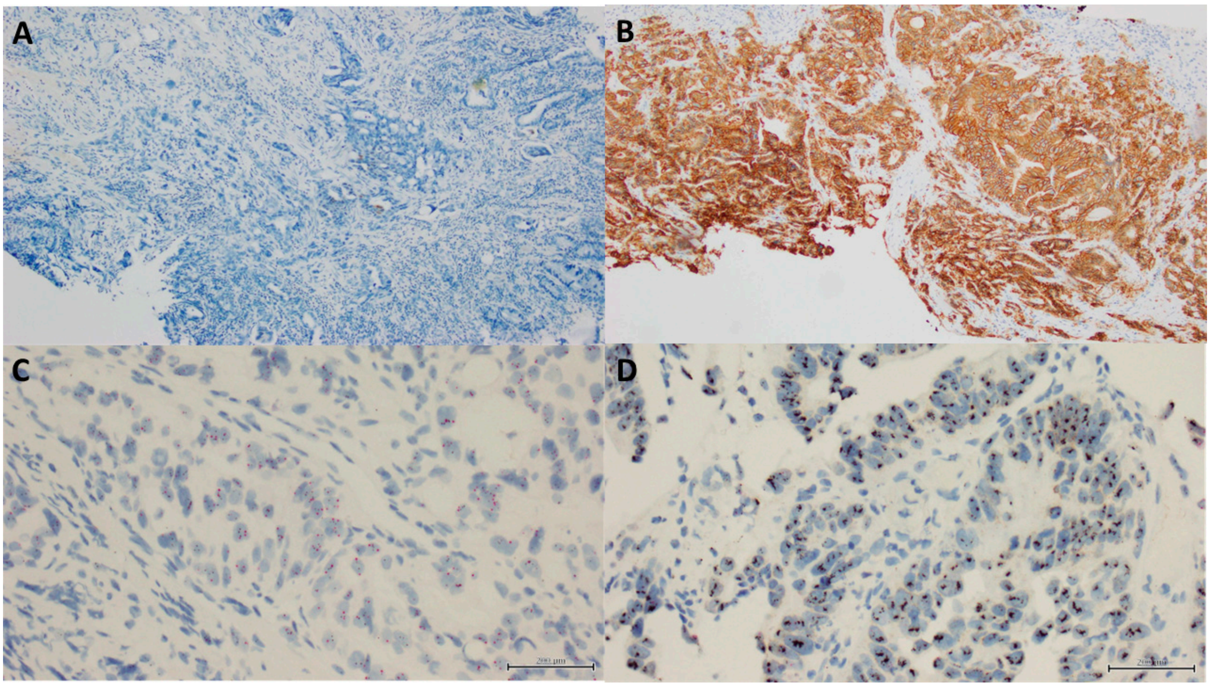

Figure S1. Examples of HER2 immunohistochemistry (IHC) and In Situ Hybridization (ISH) in gastric, gastroesophageal junction and oesophageal adenocarcinoma (at 100× magnification). (A) HER2 IHC negative (score zero). (B) HER2 IHC positive, brown staining (score 3). (C) HER2 ISH negative. (D) HER 2 ISH positive.
